# Assessment of organizational culture types, leadership styles and their relationships within governmental and non-governmental hospitals in Gaza Strip of Palestine

**DOI:** 10.1186/s12913-021-06351-1

**Published:** 2021-04-17

**Authors:** Hatem H. Alsaqqa, Çağdaş E. Akyürek

**Affiliations:** 1 Department of Health Management, Ankara University, Ankara City, Turkey; 2 Ministry of Health, Gaza City, Palestine

**Keywords:** Hospital, Leadership, Organizational culture, Palestine, Gaza Strip

## Abstract

**Background:**

The subjects of organizational culture and leadership have been studied several times in various fields. However, studies have tried to determine the relationship between corporate culture and leadership as it is still indistinguishable, or more evidence is needed. The paper describes the perceptions of the staffs about the hospitals’ organizational culture types and their managers’ leadership styles in these hospitals and the relationships that may exist between these domains.

**Method:**

This is a cross-sectional descriptive study involving 400 participants from three governmental and two non-governmental hospitals during the period from June to December 2018. The target population included all categories of staff working at hospitals as physicians, nurses, paramedics and administrators.

**Results:**

The largest number of participants was 82.5 % from government hospitals while 17.5 % were from non-governmental hospitals. Clan and hierarchy-driven cultures were the top-defined forms of organizational culture at hospitals in the Gaza Strip. In all types of organizational culture, the non-governmental hospitals which all are small size hospitals have higher perceptions’ means than the governmental ones that have different sizes. Managers’ styles in the investigated hospitals were transformational and transactional. The study’s results showed significant positive associations by Pearson’s Correlations and effect by linear multiple regression analysis between styles of transformation and transactional leaderships and types of organizational cultures.

**Discussion and conclusion:**

The study addressing the main concepts showed positive relations and also impacts between two of the leadership styles and organizational culture types, apart from the Laissez-faire style. This paper has been successful in contributing to the research on this topic and providing indications for understanding certain domains of the hospital industry in Palestine.

## Background

Management and business literature are full of research on corporate culture and leadership. In various disciplines, scholars and researchers have defined and conceptualized leadership and organizational culture (OC) in different ways. Leadership style and OC have been identified as key concepts relating to the significance of organizations. However, in the early 1970 s, efforts to explain and study leadership and culture appeared in the literature.

Leadership is fundamental to the transformation of OC [1]. Clark et al. [2] suggest that OC can be seen as a factor affecting the efficacy of an organization. This culture can be influenced and restructured by the firmly established corporate culture leader [3]. Therefore, the selection and retention of managers who can affect constructively, is an essential mission for any organization.

According to Schein [4], leadership is associated with culture creation, evolution, change and even destruction. The recruitment, selection, promotion and firing of members within an organization have a major influence on cultural growth and development. The OC is perpetuated by recruiting and retaining the members chosen by the leader. The selection of staff that corresponds to the cultural ideals and assumptions helps to embed and perpetuate culture. Current staff is usually retained and promoted based on cultural conformity criteria. Also, considering the relationship between the leader and culture is a means of realizing the organization’s functioning [4].

Cowden et al. [5] conducted a systematic review of 23 studies on management practices and the retention of nurses. They found that transformative leadership improved the retention of nurses. Likewise, Abu-AlRub et al. [6] found that Jordanian nurses proposed to remain more in their settings when democratic styles of leadership were exercised. The importance of OC underlines the need for leaders to recognize its fundamental aspects and their impacts on the results of the staff, such as the intention to remain at work [7, 8]. Healthcare organizations (HCOs) faced many challenges in the recent eras, including increasing expenses and demand for services. Although, these tasks are not exceptional to any organization and affect all organizations’ types, this leads to intricacies and variations in the environment of healthcare [9]. Giritli et al. [10] during their literature review on OC determined that leadership research studies did not provide any evocative value, without the OC was considered. Several researchers supported the correlation between transformation leadership and positive OC [10, 11].

Therefore, the leadership role is to emphasize on attention and participate in the process [12]. If leaders form culture, Schein [13] identifies these chief mechanisms for inserting culture:

•What leaders pay attention to–quantity or control.
•Leaders’ response to serious incidents.
•Resource allocation criteria.
•Role modeling, teaching and coaching.
•Detected rewards and status allocation.
•Observed recruitment criteria, selection, promotion and retirement.

According to Schein [3], cultural standards define how organizations describe leadership, how leaders generate and accomplish culture and their ability to appreciate and work with culture is one of the leaders’ talents. Leadership is primarily about creating values that inspire the staff, give meaning and instill a sense of purpose in an organization’s members. Leaders probably have a significant impact on culture, but petite work has been done to investigate this. Systems’ approaches perceive organizations as having “identity” [12]. The permanent character of the organization [14] is reflected in values, traditions, symbols, practices and the way in which the organization interprets or translates its’ environment [15]. Leaders outline identity, how the organization understands its’ exertion and its’ setting, what dealings matter, what feedback counts, what information is available, and leadership views and behaviors that are shaped by standards and limits of organizations.

In order to be an effective leader, one must be able to exactly evaluate the culture of the organization and help followers to understand it. Knowledge of OC can be an important vehicle for understanding individuals’ views and behaviors. If the beliefs of an employee are in line with the OC, the culture is considered good. When the beliefs of an employee conflict with culture, they are considered poor [16]. The success or letdown of culture can be determined by the leader and the individuals that the leader has selected to work in the system. The leader must, therefore, comprehend and diagnose the complexity and status of culture [17].

Academics who study OC as their research topic feel that OC is a complex domain. Therefore, the relationship between OC and leadership behavior has been emphasized to be integrated by different academics from various fields [18]. The Organizational Culture Assessment Inventory (OCAI) and the Multifactor Leadership Questionnaire (MLQ) questionnaires were developed and have been used in Western countries, which have very different contexts. Thus, this study attempts to determine its suitability for Palestine and Gaza Strip (GS) in particular. The authors were trying to assess these domains to make sense of what is being measured. Little research on OC and leadership using other tools or none has been conducted in the healthcare sector. There is a serious gap in the literature on healthcare management in Palestine and it is hoped that this study will help to fill in it.

However, as the purpose of this study is to assess critically the role of OC types and its relations to the leadership styles at the governmental and non-governmental hospitals in GS, it is hoped that the study may contribute to understanding the role of OC, and how it may be affected by the styles of leadership. Its’ findings will be useful for healthcare managers and policymakers to improve the strategic evaluation of healthcare provision in hospitals and may have a contribution to solve some of their stuck problems.

## The healthcare system in Palestine

According to a report by the World Health Organization (WHO) [19], the Palestinian territory’s healthcare system has three completely different political, fiscal and coordination features. It functions in the framework of political uncertainty and disputes that threaten the successful governance of the system. Also, its financial sustainability is severely limited by its dependency on donor financing, which differs according to political concerns. The fracturing of the healthcare system in Palestine at the historical, regional and organizational levels is a significant characteristic of the healthcare system [20]. This heterogeneity is difficult to be properly analyzed at both the systemic and functional levels. Thus, the Ministry of Health (MoH) opposes immense challenges in implementing comprehensive policies and integrating stakeholders [21].

MoH is the biggest human resources owner operating in the healthcare sector. In 2016, at a rate of 29.6 employees per 10,000 population, the number of employees in the Palestinian MoH raised to 14,248 employees, 52.9 % in WB and 47.1 % in GS [22]. According to the Report of the Palestinian Health Information Centre [22], general hospitals provide secondary healthcare services in different areas. Some of these hospitals are also able to provide some tertiary services. The number of general hospitals was 43 in 2016. Thirteen hospitals are controlled by MoH from the total thirty hospitals functioning in Gaza, and the rest are controlled by other entities, especially non-governmental organizations (NGOs). The MoH hierarchy categorized the hospitals into three types: 1) medical compounds containing more than one specialized hospital for Shifa and Nasir hospitals, 2) large hospitals with more than 101 beds for European Gaza hospital, Shohadaa’ Al-Aqsa hospital and Pediatric Naser hospital, 3) small hospitals with less than 100 beds for other hospitals.

## Methodology

The purpose of this study was to describe the hospitals’ employees’ perceptions of their hospitals’ OC types and managers’ leadership styles within their hospitals and to determine if there is a relationship between the two variables. OC typologies’ types and transformational leadership (TFL) and transactional leadership (TRL) styles’ ratings as perceived by employees were based upon Cameron and Quinn’s [23] cultural framework and Bass’ leadership [25; 26] theory.

A descriptive-correlational study was supposed to describe the hospitals’ staffs’ perceptions of their OC types and their managers’ leadership styles in the governmental and NGOs’ hospitals and the relationships that may exist between these two variables. Descriptive co-relational designs were used when relationships between variables are being studied and described. The study used an ex-post facto causal-comparative design. The independent variables were: TFL, TRL, laissez-faire leadership (LFL), and leadership perceived behaviors (outcomes) while the dependent variables were OC types. With this method of research design, multiple regression analysis was used to study these variables. Multiple regression is an equation based on correlation statistics in which each independent variable is entered into the equation to determine how strongly it relates to the dependent variable and how much variation in the dependent variable can be projected by each independent variable [24].

OC was designated in terms of OC sub-scale scores form the OCAI [23]. Leadership was described in terms of Bass’s [25] MLQ. The MLQ5x was used to collect data regarding the variables: TFL, TRL and LFL styles. Hospitals were described according to scores from the demographic questionnaire.

### Sampling

The target population of this study included physicians, nurses, paramedics and administrators. The authors chose different-sized hospitals for two reasons; the nature of this study; the authors decided to include participants from the different professional groups and from a cross-section of the Gaza hospitals and to ensure that the sample would be representative for all GS hospitals. Sample size is an important element in confirming that a study’s findings represent the population [26]. To determine the suitable sample size, the authors conducted a power analysis tool to ensure its findings would represent the whole population [26]. The alpha level, or error rate, was set at 0.05, as it is accepted within the social sciences field [27]. The total number of staff working in governmental and non-governmental hospitals at the time of calculating the sample was 3022. The verified sample size was 341participants and then this total sample size was increased to 400. In order to reach this sample size, the authors distributed about 470 questionnaires with a response rate 85 %.

Non-probability convenience sampling was applied for this study. However, given the dearth of information, this method was appropriate. The desired number of subjects in this sample was increased to the study design and analysis measures. The sample size was derived from a group of different employees working at different departments in governmental and NGOs hospitals in GS. The sample population was a multi-heterogeneous. The instruments to measure the variables are reliable and valid. Subjects were obtained from a population of fulltime-working employees. In sum, the research sample was selected from among the employees of governmental and NGOs hospitals by means of the following multistage sampling technique.

- Phase 1: The authors used the stratified random sampling to select the hospitals that represented the MoH (except the Psychiatric Hospital) and the NGOs hospitals in GS according to their owners and size classifications. Three governmental hospitals (compound, large and small) one from each size and two NGOs hospitals which all have the same small size type.
- Phase 2: The authors distributed questionnaires to each selected hospital according to the sample proportional distribution method.
- Phase 3: The authors distributed the questionnaires in each hospital to the professional groups according to their proportion.
- Phase 4: The authors used a non-probability convenience selection of the number required in each professional group.

Therefore, selecting the sample in this way ensured that it would represent the hospitals (the study population) and the professional groups (the target population).

### Measurement/Instrumentation

Two surveys and a demographic one were utilized as the instruments in this study. The OCAI was used to assess the OC types and the MLQ5x was used to assess the leadership styles.

### Organizational culture Assessment Inventory (OCAI)

The OCAI was developed by Cameron and Quinn [23] based on an OC framework built upon a hypothetical model referred to as the Competing Values Framework (CVF). The OCAI is used to determine the OC profile based on the core values, assumptions, and approaches that portray organizations [23]. The OCAI tool consists of six criteria, and each criterion had four substitutes, thus constructing a total of 24 items that constitute the four culture types.

**The six criteria are**:

1. ***Dominant characteristics***: the degree of cooperation and sense of belonging, the level of innovation and dynamism, the focus on goals and competitiveness, the dependence on processes and the emphasis on productivity.
2. ***Organizational leadership***: is the organization’s leadership style. The positions of leadership are established as mentor, facilitator, innovator, distributor, developer, owner, organizer and monitor.
3. ***Management of employees***: reflects how employees are handled by the corporation, the degree of consultation, engagement and consensus, and the work climate.
4. ***Organization glue***: mechanisms that hold the organization together, such as cooperation and solidarity, loyalty and dedication, entrepreneurship and versatility, rules and regulations, target alignment and competitiveness.
5. ***Strategic emphasis***: explains what drives the corporate approach (human capital’s long-term production, sustainability and competitive advantage, creativity, growth and acquisition or the accomplishment of objectives.
6. ***Criteria for success***: represents how organization describes its efficiency and how it rewards it.

**The four cultures are:**

***Clan culture***: is focused to control the environment through collaboration, engagement and unanimity and is focused on internal issues. Flexibility and discretion are favored over stability and control.

***Adhocracy culture***: is focused on external inquiries; its main values are innovation and risk taking. Stability and power, give way to versatility and discretion.

***Market culture***: is based on stability and control concepts, and external problems are of higher importance than internal issues. The external environment (market) is considered a potential threat in this community, while benefit, threat detection and competitive advantage are of supreme concern.

***Hierarchy culture***: is more subject to internal issues than with external issues, while stability and power are favored over flexibility and discretion. This culture continues to function when the organizational climate is both secure and uncomplicated and productivity is the primary objective.

The OCAI adopted normative scale as five-point Likert scale ranged from 1 = totally disagree to 5 = totally agree. Instead of the ipsative gauge or (forced-choice measurement) that used by the creating author in the original instrument that measuring the evolution of the person perception, most of the replication is applying the normative scale in their studies [28].

In addition, a demographic data questionnaire was used that described the subject sample including age, sex, educational level, work department, profession, position title and employment years at the hospital.

### Multifactor Leadership Questionnaire (MLQ)

The internationally renowned MLQ suggested by Bass [29].The MLQ is a 45-item questionnaire that measures TFL, TRL and LFL utilizing a five-point Likert scale. The MLQ measures a full range of leadership styles and behaviors including TFL, TRL and LFL and three outcomes of leadership style: extra effort; effectiveness and satisfaction. The MLQ5x is both a self-report and rater-report measure of leadership style and leader behaviors based on Bass’s [30] theory of TFL and TRL. Strong evidence supports the validity and reliability of the current version of the MLQ Form 5X [31]. This study analyzed the perceptions of the hospitals’ staffs, therefore, only the rater form was used. Raters complete the MLQ5x to evaluate how frequently or to what degree, they are able to observe the leader display or take part in specific behaviors and the additional leadership outcomes’ items. The aspects for TFL are idealized attributes, idealized behavior, inspirational motivation, intellectual stimulation, and individualized consideration. The TRL aspects are contingent reward and management by exception, active and passive, however LFL measures non-leadership.

According to Schwartz et al. [32], the three leadership styles have different characteristics as follow:

- *Transformational leadership style*: shows trust, generates a common sense of purpose, explores innovative approaches to problem solving, serves as a mentor and instructor, takes the individual and their needs into account.
-*Transactional leadership style*: is focused on a mutual exchange between the leader and the followers. This style includes offering workers anything in exchange for their compliance with and recognition of authority (e.g., incentives, pay raises, status increases).
- *Laissez-faire leadership style*: avoids involvement in significant problems, is absent when needed, avoids decision-making and delays in responding to crisis cases.

### Instrument translation method

The instruments used in this study had been originally developed in English and were translated into Arabic for use with other researches. The authors preferred to retranslate the questionnaires depending on the original questionnaires to avoid any misinterpretation in the meaning of each item and to be more comfortable with the Palestinian context. The experts used the already translated Arabic versions as assistant materials. A standard three-step protocol reported by Blaschko and Burlingame [33] was used when translating the questionnaires. Back translation is a common technique involving translating the items of a questionnaire from the source language to the target language.

### Pilot study

The authors used the pilot study mainly to reflect the reliability and validity of the Arabic translated version of the two used questionnaires. The pilot study was done in one of the hospitals that excluded by the sampling process from the data collection of this research. Thirty respondents from one of the non-chose hospitals’ in the sample were selected to take part in the study to examine their responses to the questionnaires, to explore the appropriateness of the study instruments, the language and the understanding of the items. Most of the respondents had no problem understanding the two questionnaires’ parts. The authors conducted Cronbach alpha internal consistency coefficient test for measuring the reliability of the two questionnaires. It was found to be 0.937 and 0.932 for the OCAI questionnaire and the MLQ5x questionnaire consequently.

### Data collection

A cover letter and instructions packet directed to the governmental and NGOs hospitals’ participants, the MLQ, OCAI, and demographic questions were accompanied. The cover letter encompassed the following information: (i) the aim of the questionnaire, (ii) an estimate of the time it was expected that participants would require to answer the questions and (iii) a statement guaranteeing anonymity and confidentiality for the participants.

In June 2018, after obtaining the approval of the hospitals to conduct the study, the authors delivered all questionnaires to the participants by a data collection team. The authors followed up the process to increase the response rate as believing that participants were likely to feel under no obligation to be involved in the study. The data collection was completed by December 2018. Ethical and administrative considerations and procedures were considered.

### Data entry and analysis

Overviewing of the surveys was the first step preceding the data entry. This step tracked by scheming an entry model using the computer software Statistical Package for Social Sciences (SPSS) version 22. Then, the coded variables entered into the computer by the authors. Data entry conducted in congruency with the data collection. Samples of entered questionnaires were checked followed by conducting frequency distribution to check the accuracy, missing values and conduct data cleaning.

### Descriptive statistics

#### Hospitals’ and participants’ characteristics

The study included 5 hospitals; 3 governmental and 2 NGOs hospitals in the GS to ensure reflecting the various cultures existing at the different hospitals. The demographic analysis represented about 60 % of the sample was male and 40 % was female. Most of the participants’ age ranged between 20 and 40 years. This age group that was known as a young adult started with identity development, ready to familiarity and sympathy. Young adults are highly inspired capable of self-efficacy, which lead to accomplishing the work requirements.

Regarding the work profession, nursing had the highest percentage with 37.2 %, physicians with 28.8 %, administrators had 19.0 % and paramedics constituted 15 %. The compound hospitals’ participants represented 55.3 % while the small hospital participants had 26.4 % with only 18.3 % for the large hospitals. This indicates that the authors included different staff categories within the different hospitals to ensure the multiplicity of the cultural views and to determine the discrepancy between categories and to reflect the various numbers of participants in each hospital according to its capacity (according to the MoH classification). These points can shed the light to the different manners and tools that may prerequisite to deal with the different categories, which profession has the indigence to start the improvement with, and where the focus of efforts has to be amplified.

Of the participants, 65.1 % had between 1 and 10 work years in hospitals. Approximately 81 % had 6 years or more of working in the healthcare sector. Around 71 % of them were ordinary employees while nearly 21 % were sections or departments’ heads; the rest were having other different positions. Thus, the working staff in Gaza hospitals may have different knowledge about the hospitals’ cultures and various awareness of the leadership styles that drive them.

#### Hospitals’ organizational cultures types’ and leadership styles’ dimensions

To clarify the overall picture of hospitals’ OC, the authors obtained cultural profiles for the hospitals by examining the respondents’ ratings means for the four OC types. The authors found that the perceptions of OCs’ dimensions were the highest with the clan culture, hierarchy culture and adhocracy culture with 3.36, 3.27 and 3.24 respectively. The six content areas also demonstrated a congruent OC as perceived by the participants. Cultural congruence indicates that the diverse components of an organization’s culture are aligned [34]. In other words, in four of the six content areas, clan culture predominates. These results go with another study aimed to identify the major culture within the Palestinian Primary Healthcare Centers of the MoH and the Centers of the United Nations Relief and Works Agency for Palestine Refugees (UNRWA) by using the CVF [35]. Contra-indicatory to our results, the authors resolved that in private Jordanian hospitals, hierarchy culture was the foremost, clan culture was the least dominant [36], while market and adhocracy cultures were found at an intermediate level. However, this result may coincide with the context in the private sector, which is not covered in this study.

Moreover, the analysis of the data showed that there were no statistically significant differences between organization culture types and sex, age, educational level and employment years in hospitals. Regarding the OC types with the different professions of the participant, the study presented that the paramedics had higher perception mean than the other professions with all types of culture (p-value = 0.00), while the physicians had the lowest mean with all culture types. The differences were between nurses, physicians, and paramedics as showed by Post Hoc-Scheff test. The departments’ heads perceived the lowest means for clan, hierarchy, adhocracy cultures in their hospitals. The authors denote to the awareness level and high expectancy from the departments’ heads toward the OC concept that may affect their evaluations negatively.

To examine the differences in the OC types’ means in reference to the hospitals’ owners, an independent t-test was used to compare the means of the 4 culture types of OCAI. According to the types of hospitals’ owners, there were significant differences between the governmental and NGOs hospitals for all culture types, but the highest means were for the NGOs with the clan and hierarchy culture with 4.12 and 4.02 respectively at *p*-value = 0.00. The authors suggest that the general perceptions about culture types in the NGOs were higher than the governmental ones because of its small size that made culture types more perceptual for hospitals’ staff. Generally, small to medium-sized organizations are rigid in mindset and are also controlled by the owner or the founder.

OC research measured by various groups of healthcare workers will help administrators decide if there are variations in the attitude of staff groups in hospitals. On that basis, the leader should formulate the most suitable approaches, which would focus on specific groups to achieve consistency in the desired OC pattern. It also implies that the leaders can handle human resources efficiently in general. The managers can be better equipped to identify policies that have a great impact on the employees of the hospitals in each target group.

Regarding the MLQ5x rater form, which analyzed three leadership styles and three leadership outcomes. The laissez-faire leadership style had the lowest mean (M = 3.26, SD = 0.92), while the TFL style had the highest mean (M = 3.68, SD = 0.57) followed by TRL (M = 3.50, SD = 0.47). The outcomes had the means M = 3.62, 3.64 and 3.68 for extra-effort, effectiveness and satisfaction respectively. The results from the descriptive figures indicated that the participants sometimes to fairly-often supposed the leadership as having a mixture of TFL (team-oriented) and TRL (task-focused) styles, with LFL being shown occasionally. In addition, leadership was supposed to frequently display extra-effort, effectiveness and satisfaction. These leaders get staff members to do more than what are expected, work satisfactory with others and are effective in meeting job-related requirements. In Saudi Arabia, Omer [37] and Abu-AlRub et al.. [6] found the same results as both nurses’ managers and staff gave a higher rating to transformational than transactional behaviors. The respondents alleged the leadership style of their managers as TFL at a moderate-high level. In the selected hospitals, both TFL and TRL styles are blended. This result is consistent with Bass [38], who stated that the transformational and transactional approach of leadership can be applied at the same time.

T-test was applied to differentiate between the leadership styles and outcomes regarding the participants’ sex. The data showed that there were no statistically significant differences between leadership styles except for TFL where male had higher mean than female. However, for all leadership outcomes there were statistically significant differences according to the sex. Also, the data showed that there were no statistically significant differences between leadership styles and also between outcomes regarding the age and the educational level factors. In addition, regarding the participants’profession, there were differences regarding the TRL only where the nurse and paramedic professions had the higher means. Using Scheff-test showed the differences were between doctor and paramedic categories. Regarding the position title the differences were only for LFL, with the departments’ heads scored the higher mean. However, the authors pointed out that the profession and work position variables had not reflected differences because the assessment was related to the strategic level of the hospitals’ leaderships. Consequently, all employees’ categories and positions focus on the organizational-level management of the hospitals so the internal integrity and span of control may be perceived roughly the same. Concerning the employment years at hospitals, only the effectiveness outcome had differences between the participants with higher mean gained by the newest work group (1–5 years).

There were statistical differences between hospitals’ sizes regarding the TRL and LFL. The small hospitals had the highest means with TRL (M = 3.62). On the other hand, the compound hospitals had the lowest mean with LFL (M = 3.46). The Post-hoc test showed that the differences were between compound and large hospitals for the TRL and between the compound and small hospitals for the LFL. Though, the NGOs hospitals had the highest means with the TFL and TRL (M = 4.00 and M = 3.84 respectively at *p*-value = 0.00). The authors explained that NGOs hospitals were small hospitals therefore management style in small settings generally is more explicit than the larger ones. Nonetheless, the role of leadership in small hospitals can be perceived and investigated more than large and compound hospitals.

To present the relation between the OC and the leadership, Pearson’s correlations were performed for the three leadership styles (transformational, transactional and laissez-faire) on each of the four recognized OC types to determine whether there were relationships between these variables. These relationships specify the strength and direction of the associations. The results are shown in Table 1.

Significant, positive and moderate-level relationships were found between the TFL and clan (*r* = 0.403), adhocracy (*r* = 0.404), market (*r* = 0.389) and hierarchy cultures (*r* = 0.395) (*p*-value < 0.05). Similarly, it shows significant and positive low-level relationships between the TRL and clan culture (*r* = 0.173), adhocracy (*r* = 0.208), market (*r* = 0.186) and hierarchy cultures (*r* = 0.183) (*p*-value < 0.05). In addition, there were no significant relationships between the LFL and OC types (*r* = -0.014, *r* = -0.047, *r* = -0.048 and *r* = -0.028 respectively).

**Table 1:** Pearson’s Correlations Between the Leadership Styles and Organizational Culture Types

Variables	Clan	Adhocracy	Market	Hierarchy
**Transformational**	0.403*	0.404*	0.389*	0.395*
**Transactional**	0.173*	0.208*	0.186*	0.183*
**Laissez-Faire**	-0.014	-0.047	-0.048	-0.028

Results of the study addressing the main questions showed significant positive correlations between two of the leadership styles and OC types, apart from the LFL style, which is not significantly correlated with any culture type. Laissez-Faire is the utmost passive leadership style as it refrains from responsibilities, entrusts tasks to others, is absent in critical situations, and has a detached character in controlling the concerns and the conditions governing the organization. Therefore, it does not have a substantial consequence on their followers’ perceptions of OC. The main results suggest that the responses from the participants will likely to be in the same direction for both TFL and TRL leadership styles with OCs. In other words, TFL and TRL leadership styles are significantly correlated to OCs.

To evaluate the significance of the relationships between the three leadership styles and OC types, additional analyses were conducted using linear multiple regressions. It was observed that the calculated VIF values were between 1.450 and 2.290 and the tolerance values were between 0.768 and 0.934. Thus, it was concluded that there was no multiple dependency problem and the reliability of the model was high as Durbin-Watson value is between 1.5 and 1.6.

According to the results of the regression analysis, it was indicated that the transformational and transactional leadership styles had significant effects on clan culture (β = 0.283) and (β = 0.125) at *p*-value < 0.05. It was also presented that transformational leadership style and transactional leadership style had significant effects on adhocracy culture (β = 0.309) and (β = 0.129). The results also explained that transformational leadership style and transactional leadership style had significant effects on market culture (β = 0.273) and (β = 0.143). Nonetheless, transformational leadership style and transactional leadership style had significant effects on hierarchy culture (β = 0.261) and (β = 0.145). The laissez-faire leadership style had not any significant effect on any culture type (*p*-value >0.05).

In conclusion, the regression analysis showed that the OC types can be affected by the leadership styles with a significant relationship (F(3.396) = 18.977) for clan culture, (F(3.396) = 22,881) for adhocracy culture, (F(3.396) = 19.688) for market culture and (F(3.396) = 18.276) for hierarchy culture. However, the leadership styles all together disclose 12.6 % of clan culture and 14.8 % of adhocracy culture and 13.0 % of market culture and 12.2 % of hierarchy culture (Table 2.). This result is consistent with the conclusions of Tsai [11], which indicate that because the culture of any organization is progressively framed through its leaders; therefore, uniformity between TFL behaviors and the alleged culture by followers become solid and strong by time.

Cameron and Quinn [23] proposed that a TFL’s behaviors such as encouraging inspiration would be found in clan cultures and this study provides robust evidence to support that hypothesis. There were also noteworthy relationships between TRL and adhocracy culture that is contrary to what Cameron and Quinn predicted, whereas transactional performance would be found in rule-based culture. Therefore, respondents want their representatives to consider their staffs’ needs and expectations in order to explain and inspire them and also create sound internal policies that encourage the staff to work and improve the hospitals’ competitiveness. In addition, managers need to incorporate more innovative and creative approaches to improve hospital productivity and capacity, particularly when the healthcare sector is evolving into full autonomy and global integration.

**Table 2:** Regression of Leadership Styles on Organizational Cultures Types

Variable	B	SE	β	t	P	VIF
Clan Culture						
Constant	1.288	0.321		4.010	0.000	
Transformational	0.398	0.074	0.283	5.409	0.000*	1.902
Transactional	0.203	0.087	0.125	2.326	0.021*	2.290
Laissez-Faire	-0.026	0.044	-0.029	-0.605	0.546	1.450
*R* = 0.355 *R*² =0.126 F (3,396) = 18.977 *p* = 0.000 Durbin-Watson = 1.5						
Adhocracy Culture						
Constant	1.180	0.306		3.851	0.000	
Transformational	0.419	0.070	0.309	5.967	0.000*	1.902
Transactional	0.204	0.083	0.129	2.443	0.015*	2.290
Laissez-Faire	-0.054	0.042	-0.062	-1.296	0.196	1.450
*R* = 0.384 *R*² =0.148 F (3.396) = 22.881 *p* = 0.000 Durbin-Watson = 1.6						
Market Culture						
Constant	1.244	0.311		3.994	0.000	
Transformational	0.373	0.071	0.273	5.224	0.000*	1.902
Transactional	0.226	0.085	0.143	2.668	0.008*	2.290
Laissez-Faire	-0.059	0.042	-0.068	-1.396	0.163	1.450
*R* = 0.360 *R*² =0.130 F (3.396) = 19.688 *p* = 0.000 Durbin-Watson = 1.5						
Hierarchy Culture						
Constant	1.170	0.335		3.490	0.001	
Transformational	0.382	0.077	0.261	4.966	0.000*	1.902
Transactional	0.247	0.091	0.145	2.708	0.007*	2.290
Laissez-Faire	-0.046	0.046	-0.049	-1.002	0.317	1.450
*R* = 0.349 *R*² =0.122 F (3.396) = 18.276 *p* = 0.000 Durbin-Watson = 1.5						

*p < 0.05

## Discussion

The study was intended to fill a gap in the literature concerning the OC types and leadership styles that are being applied in the GS hospitals. As the hospital setting is the major provider of healthcare, it can be assessed as a mean of defining how other organizations in the sector are proceeding. For that, the authors conducted this study in five hospitals at both governmental and NGOs sectors. In doing so, the authors hoped to create perspectives that would help executives, students, managers and policymakers to address their challenges. The findings of this study may be relevant to all stakeholders because the literature indicates that leaders affect OC [39] by generating confident and fruitful change within organizations [34].

More specifically, it is hoped that the study may contribute to understanding the role of OC in healthcare provision and also to fill a serious breach in the literature of HCOs management in Palestine. More widely, the study contributes to scant the literature on the relationship between the OC types and leadership styles on managerial behavior. The findings of this study showed that hospitals were not characterized by just one cultural type, although in this assessment the clan culture and hierarchy culture were marginally dominant. This was followed by the adhocracy and market types in this order. Generally, most hospitals being characterized by a high level of bureaucracy and driven by rules and regulations. Also, the three leadership styles and outcomes that are founded in the investigated hospitals, predominated by the TFL, TRL with satisfaction and effectiveness that scored the highest outcomes.

The results revealed that OC in all hospitals included in this study was clan and hierarchy culture (internal focus). These cultures in the governmental hospital were not outlandish. As maintained in the literature that both bureaucratization and family climate existence in the public organization are high. That led to a high consistent product, services and efficiency, with a stable environment that leads to uniformity of services maintaining. These values motivate staff to show respect to their superiors and accept the power distance between superiors and subordinates. The similarity in the scores’ levels in most of the dimensions revealed the cultural situation that was already present in GS hospitals. This implicates the interdependency the dimensions have over others. So, when OC initiative or approach must be more comprehensive and multidimensional one so that incrementally parallel positive effects will actually change the direction of the hospitals’ OCs.

Moreover, the statistical findings of the variables examined in the study showed that the mean scores tended towards responses of ‘fairly often’ and ‘frequently’ from the respondents due to the perceived or observed behaviors of the leaders being rated. This study provided insight into the nature of leadership styles and extends our understanding of the association between TFL, TRL and LFL styles and leadership outcomes. Overall conclusions of the study supported the propositions of the Full Leadership Model that TFL extends the results of TRL toward results beyond expectations. In line with our observations and results from previous research [40, 41], we can accomplish that TFL can make a significant contribution to the organizations’ effectiveness and that there is a need for further leadership styles examination at diverse levels.

The results showed that there were significant relationships between the three leadership styles identified and the three outcomes from the MLQ5x. Leadership and OC are two key factors in determining organizational outcomes and understanding how these concepts affect each other is crucial for optimal organizational success. The prominent most vital finding of this research was that there is a significant positive correlation between both TFL and TRL in all culture types but not for LFL. This approach will serve as an important guide for leaders from organizations who support matching leadership style with OC. It appears that the hospitals need to lessen the internal controls and follow more dynamic management and entrepreneurial and result-oriented leadership.

The study also presented that the leadership styles had significant influences on all culture types except for the LFL style. Indeed, the strength of this influence may prove to be integral to the problems and challenges facing the HCOs. Although not all leadership styles demonstrated an impact on the OC types, the data showed that participants of hospitals in this study seemed to have reasonable expectations of their managers.

As a result of the ambiguity of the OC and its’ measurability, it is rarely considered or used by the policymakers either in planning or their visions. That leads to the lack of evidence of its’ importance or effective role in HCOs. This study’s results make informing of organizational evaluations and support management decision-making regarding the implementation of leadership styles and management models in hospitals. It is also relevant from an academic opinion, as it contributes to endorse precise knowledge within an area in which empirical studies are still scarce.

### Study limitations

The study findings should be interpreted in consideration of several limitations. Firstly, the study employed a convenience sampling technique for the data collection from the different staff categories, which is a non-probability sampling method that might have affected the generalizability of the results. The cross-sectional design of the study had some weakness as it is liable to contextual changes. Secondly, as the OC and leadership in hospitals are sensitive areas in its nature, hospitals’ staffs would be more likely to underestimate or over report their responses. Another concern is related to a measure of OC and MLQ5x by self-report of respondents. Thirdly, the limited financial and time resources available, the empirical part of this research was conducted exclusively among the governmental and NGOs hospitals in GS.

## Conclusions

The study reveals that there was an agreement between the results of the dominated culture types and leadership styles. The clan and hierarchy cultures were slightly prevalent in this assessment however, most hospitals are characterized by a high level of bureaucracy and are driven by rules and regulations. This study affords evidence that leadership behavior and values affect not only their activities and decisions but also the culture of the organizations they lead. Consequently, the personality of top-level leaders is felt throughout the organizations by influencing the types of people who join and endure with the organizations, norms that authorize or discourage members’ behavior and decision making and also interaction among members. There is also an increase in supporting evidence that demonstrates effective leadership as an integral part of organizational effectiveness [42, 43].

Identifying and implementing the most effective leadership style is critical to the transformation of GS hospitals sector. Selecting effective leadership styles and approaches will guarantee that managers are well educated and controlled. The transformational leadership style was found to be the most effective in achieving the outcomes of staff satisfaction and willingness to apply extra-effort. Researchers could empirically and clinically monitor the phases that organizations’ founders and leaders have to take to integrate their characteristics and ideas into the administrations they control.

### Implications for practice and research

The author considered some of the implications for further studies of OC and leadership in hospitals and healthcare settings; (1) using a longitudinal perspective will help to identify the details in the culture formation procedure. This suggests that leaders may play a critical role in the accomplishment or disappointment of organizational change and development enterprises; (2) further research should be developed to understand the values and human relationship factors contributing to promote a healthy relationship between the leadership styles and the OC types; (3) while the findings of this study have donated to the knowledge regarding the topic, a detailed qualitative study, with an emphasis on cause and consequence, might provide a deeper thoughtful of the variables in this study; (4) research could be done to study the relationships between leadership, culture, and other factors related to hospitals such as regions, workloads, beds occupancy rate and hospitals conformity to standards in a matrix approach; (5) further research could be performed by comparing OC types and leadership styles with different healthcare settings, as in the private sector and primary healthcare centers.

Concisely, the findings of this study encourage more studies to be specified to populations of different care units and within dissimilar healthcare institutions in the GS context. Thus, the replication of this study to healthcare settings within Palestine and the Middle East Region can be complementary and instructive to the generalizability of the study’s tangible outcomes.

## Data Availability

The dataset was saved with the correspondent author in a proper and reusing possibility manner. The right to accessibility and review of the data can be provided at any time in case of an inquiry.

## Abbreviations

OC: Organizational culture
OCAI: Organizational Culture Assessment Inventory
CVF: Competing Values Framework
MLQ: Multifactor Leadership Questionnaire
NGOs: Non-governmental organizations
MoH: Ministry of Health
GS: Gaza Strip
TFL: Transformational leadership
TRL: Transactional leadership
LFL: Laissez-faire leadership
